# Supplementary material for: Native Plant Growth-Promoting Rhizobacteria Containing ACC Deaminase Promote Plant Growth and Alleviate Salinity and Heat Stress in Maize (Zea mays L.) Plants in Saudi Arabia
Source: Plants (Basel). 2025 Apr 2;14(7):1107. doi: 10.3390/plants14071107 (PMC11991235; doi:10.3390/plants14071107)
Supplement: Supplementary file 1 [file plants-14-01107-s001.zip › plants-3524131-supplementary.docx]

**Table S1.** Characterization of the ability of sixty-six bacterial isolates to grow at two different temperatures (i.e. 45̊ C and 50̊ C).

| Strain code | Identification based on 16S rRNA sequencing | 45 ± 0.1°C | 50 ± 0.1°C |
| --- | --- | --- | --- |
| SFO16 | *Psychrobacter faecalis* | - | - |
| SFO021 | *Bacillus subtilis* | + + + | + + + |
| SFO033 | *Staphylococcus paralicheniformis* | + | + |
| SFO037 | *Bacillus paramycoides* | - | - |
| SFO040 | *Paenibacillus lautus* | + + | - |
| SFO041 | *Arthrobacter crystallopoietes* | - | - |
| SFO043 | *Cytobacillus oceanisediminis* | + + + | + + |
| SFO059 | *Ensifer adhaerens* | + | + |
| SFO063 | *Bacillus haikouensis* | - | - |
| SFO074 | *Kocuria palustris* | - | - |
| SFO075 | *Salinicola halophilus* | - | - |
| SFO085 | *Nocardioides luteus* | - | - |
| SFO091 | *Staphylococcus pasteuri* | - | - |
| SFO098 | *Staphylococcus pasteuri* | - | - |
| SFO102 | *Bacillus siamensis* | + + | - |
| SFO106 | *Bacillus velezensis* | + + | + |
| SFO109 | *Bacillus anthracis* | + | + |
| SFO112 | *Staphylococcus pasteuri* | + + + | + + + |
| SFO119 | *Bacillus inaquosorum* | + + + | + |
| SFO120 | *Priestia koreensis* | - | - |
| SFO121 | *Metabacillus indicus* | + + + | + + + |
| SFO132 | *Staphylococcus petrasii* | - | - |
| SFO133 | *Staphylococcus pasteuri* | + + + | + + + |
| SFO134 | *Staphylococcus pasteuri* | + + | + + |
| SFO144 | *Bacillus velezensis* | + + + | + |
| SFO145 | *Bacillus haynesii* | + + + | + + + |
| SFO152 | *Stutzerimonas stutzeri* | - | - |
| SFO158 | *Bacillus velezensis* | + | + |
| R600 | *Pseudomonas soyae* | - | - |
| R900 | *Pseudomonas canavaninivorans* | - | - |
| R114 | *Paenarthrobacter nitroguajacolicus* | - | - |
| R127 | *Paenarthrobacter nitroguajacolicus* | - | - |
| R142 | *Pseudomonas koreensis* | - | - |
| R160 | *Pseudomonas guariconensis* | - | - |
| Z117 | *Bacillus wiedmannii* | ++ | - |
| Z180 | *Bacillus spizienii* | + + + | + + + |
| Z183 | *Streptomyces venezuelae* | - | - |
| B200 | *Bacillus licheniformis* | + + + | + |
| B1105 | *Arthrobacter cheniae* | + + + | + + |
| K1 | *Enterobacter cancerogenus* | - | + |
| K2 | *Bacillus stercoris* | + + + | + |
| K3 | *Microbacterium maritypicum* | - | + |
| K4 | *Staphylococcus hominis* | + | + |
| K5 | *Klebsiella oxytoca* | - | + |
| K6 | *Bacillus licheniformis* | - | - |
| K8 | *Acinetobacter calcoaceticus* | - | + |
| K9 | *Pseudomonas lini* | - | + + |
| K10 | *Chryseobacterium rhizoplanae* | - | + + |
| K11 | *Pseudomonas frederiksbergensis* | - | - |
| K12 | *Stenotrophomonas tumulicola* | - | + + |
| K13 | *Staphylococcus pasteuri* | - | + + |
| K14 | *Stenotrophomonas tumulicola* | - | + |
| K16 | *Achromobacter spanius* | - | + |
| K17 | *Paenibacillus sp.* | - | + |
| K18 | *Sphingobacterium prati* | - | - |
| K19 | *Pseudomonas frederiksbergensis* | - | - |
| K20 | *Cellulomonas pakistanensis* | - | - |
| K21 | *Staphylococcus pasteuri* | - | - |
| K22 | *Bacillus paralicheniformis* | + + + | + + + |
| K23 | *Stenotrophomonas indicatrix* | - | - |
| K24 | *Paenibacillus lautus* | + + + | - |
| K25 | *Pseudomonas frederiksbergensis* | - | - |
| K26 | *Pseudomonas thivervalensis* | + + + | + + |
| K27 | *Chryseobacterium lathyri* | + + | - |
| K28 | *Bacillus paralicheniformis* | + + + | + + + |
| K29 | *Pseudomonas frederiksbergensis* | - | - |

Where; ‘-’ = highly sensitive, ‘+’ = relatively low tolerant, ‘++’ = relatively medium tolerant, and ‘+++’ = relatively highly tolerant.

**Table S2.** Qualitative characterization of the salt tolerance ability of Sixty-six isolates at three different salinity levels (2.5% NaCl, 5% NaCl, and 10% NaCl).

| Strain code | Identification based on 16S rRNA sequencing | NaCl 2.5% | NaCl 5% | NaCl 10% |
| --- | --- | --- | --- | --- |
| SFO16 | *Psychrobacter faecalis* | + + + | + + + | + |
| SFO021 | *Bacillus subtilis* | + + + | + + + | + + + |
| SFO033 | *Staphylococcus paralicheniformis* | + + + | + + + | - |
| SFO037 | *Bacillus paramycoides* | + + + | + + + | + + + |
| SFO040 | *Paenibacillus lautus* | + + | + | - |
| SFO041 | *Arthrobacter crystallopoietes* | + + + | + + | - |
| SFO043 | *Cytobacillus oceanisediminis* | +++ | +++ | ++ |
| SFO059 | *Ensifer adhaerens* | ++ | + | + |
| SFO063 | *Bacillus haikouensis* | +++ | +++ | +++ |
| SFO074 | *Kocuria palustris* | +++ | +++ | ++ |
| SFO075 | *Salinicola halophilus* | +++ | +++ | +++ |
| SFO085 | *Nocardioides luteus* | +++ | +++ | ++ |
| SFO091 | *Staphylococcus pasteuri* | +++ | - | - |
| SFO098 | *Staphylococcus pasteuri* | +++ | ++ | - |
| SFO102 | *Bacillus siamensis* | +++ | +++ | +++ |
| SFO106 | *Bacillus velezensis* | +++ | ++ | + |
| SFO109 | *Bacillus anthracis* | +++ | +++ | - |
| SFO112 | *Staphylococcus pasteuri* | +++ | +++ | +++ |
| SFO119 | *Bacillus inaquosorum* | + + + | + + + | + + |
| SFO120 | *Priestia koreensis* | + | - | - |
| SFO121 | *Metabacillus indicus* | + + + | + + + | + + + |
| SFO132 | *Staphylococcus petrasii* | + + + | + + + | + + + |
| SFO133 | *Staphylococcus pasteuri* | + + + | + ++ | + + + |
| SFO134 | *Staphylococcus pasteuri* | + + + | + + + | + + + |
| SFO144 | *Bacillus velezensis* | + + + | + + + | + + |
| SFO145 | *Bacillus haynesii* | + + + | + + + | + + + |
| SFO152 | *Stutzerimonas stutzeri* | + + + | + | - |
| SFO158 | *Bacillus velezensis* | + + + | + + + | + + + |
| R600 | *Pseudomonas soyae* | + + | + + | - |
| R900 | *Pseudomonas canavaninivorans* | + + | + + | - |
| R114 | *Paenarthrobacter nitroguajacolicus* | + + | + + | + |
| R127 | *Paenarthrobacter nitroguajacolicus* | + + + | + + | + |
| R142 | *Pseudomonas koreensis* | + + + | + + | + |
| R160 | *Pseudomonas guariconensis* | + + | + + | + |
| Z117 | *Bacillus wiedmannii* | + + + | + + + | + |
| Z180 | *Bacillus spizienii* | + + | + + | + |
| Z183 | *Streptomyces venezuelae* | + + + | - | - |
| B200 | *Bacillus licheniformis* | + + + | + + + | + + + |
| B1105 | *Arthrobacter cheniae* | + + + | + + + | + + + |
| K1 | *Enterobacter cancerogenus* | + + + | + + | - |
| K2 | *Bacillus stercoris* | + + + | + + | - |
| K3 | *Microbacterium maritypicum* | + + | + | - |
| K4 | *Staphylococcus hominis* | + + + | + + + | + + + |
| K5 | *Klebsiella oxytoca* | + + | + | - |
| K6 | *Bacillus licheniformis* | + + + | ++ | + |
| K8 | *Acinetobacter calcoaceticus* | + + + | + | - |
| K9 | *Pseudomonas lini* | + + | + | - |
| K10 | *Chryseobacterium rhizoplanae* | + + | + | - |
| K11 | *Pseudomonas frederiksbergensis* | + + | + | - |
| K12 | *Stenotrophomonas tumulicola* | + + | + | - |
| K13 | *Staphylococcus pasteuri* | + + | + | - |
| K14 | *Stenotrophomonas tumulicola* | + + + | + + | + |
| K16 | *Achromobacter spanius* | + + | + | + |
| K17 | *Paenibacillus sp.* | + + | + | - |
| K18 | *Sphingobacterium prati* | + + + | + | - |
| K19 | *Pseudomonas frederiksbergensis* | + + + | + + | - |
| K20 | *Cellulomonas pakistanensis* | + + + | + + | - |
| K21 | *Staphylococcus pasteuri* | + + + | + + | - |
| K22 | *Bacillus paralicheniformis* | + + + | + + + | + + + |
| K23 | *Stenotrophomonas indicatrix* | + + | + | - |
| K24 | *Paenibacillus lautus* | + + + | + + | - |
| K25 | *Pseudomonas frederiksbergensis* | + + + | + + | - |
| K26 | *Pseudomonas thivervalensis* | + + + | + + | - |
| K27 | *Chryseobacterium lathyri* | + + + | + + + | + |
| K28 | *Bacillus paralicheniformis* | + + + | + + + | + + + |
| K29 | *Pseudomonas frederiksbergensis* | + + + | + + | + |

Where; ‘-’ = highly sensitive, ‘+’ = relatively low tolerant, ‘++’ = relatively medium tolerant, and ‘+++’ = relatively highly tolerant.

**Table S3.** Physio-chemical properties of soils from which bacteria were isolated.

| Unit | Accacia isolates | Mangrove isolates | Olive isolates |
| --- | --- | --- | --- |
| pH | 7.78 | 8.7 | 7.67 |
| EC (dS/m^-1^) | 1.82 | 19.24 | 2.03 |
| OM% | 0.81 | 1.87 | 0.92 |
| CaCO_3_ | 32 | ND | 8.41 |
| Total N% | 0.04 | 1.06 | 0.07 |
| P mg Kg^-1^ | 5.78 | 19 | 6.46 |
| K mg Kg^-1^ | 172 | 182 | 240 |
| Texture | Sandy Loam | Loamy Sand | Sandy Loam |
| Sampling location | Huraymila, near Riyad, central Saudi  Arabia | The Arabian Gulf shore | Al Jouf Region, North of Saudi Arabia |

Mean Values of Physio-chemical properties.

**Table S4.** IAA production of selected PGPR isolates.

| Serial# | Isolates | IAA (μg ml^-1^) |
| --- | --- | --- |
| 1 | SFO075 | 2.26±1.54 |
| 2 | SFO085 | 17.80±2.67 |
| 3 | SFO091 | 39.50±6.54 |
| 4 | SFO098 | 36.15±2.53 |
| 5 | SFO132 | 7.07±1.75 |
| 6 | SFO145 | 4.39±2.86 |
| 7 | K2 | 3.46±6.36 |
| 8 | K21 | 19.80±0.92 |
| 9 | K22 | 20.82±3.87 |
| 10 | Z180 | 33.18±1.54 |
| 11 | R600 | 5.65±2.39 |


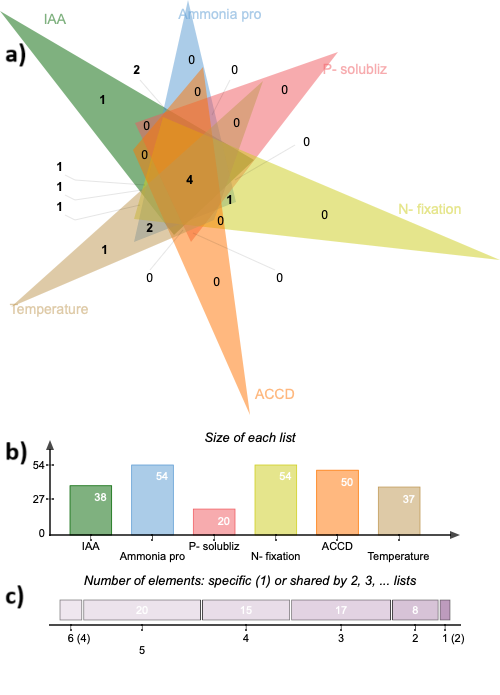


**Figure S1.** Thermotolerant bacterial isolates with multiple plant growth-promoting (PGP) activites. The possible combinations for a) five different PGP evaluated traits in addition to heat (50 ̊C) tolerance, b) size of each list of studied traits, and C) number of isolates specific to sharing each of the 6 traits.


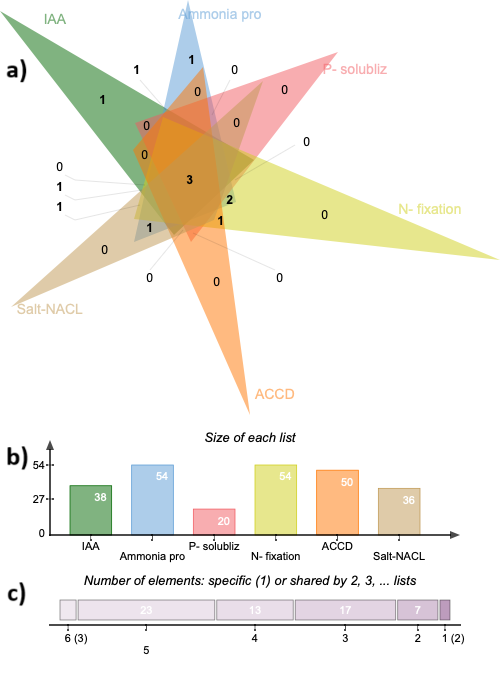


**Figure S2.** Halotolerant bacterial isolates with multiple plant growth-promoting (PGP) activites. a) five different PGP evaluated traits in addition to salinity tolerance, b) the size of each of the PGP traits and salt-NaCl, and c) number of isolates specific to sharing each of the 6 traits.


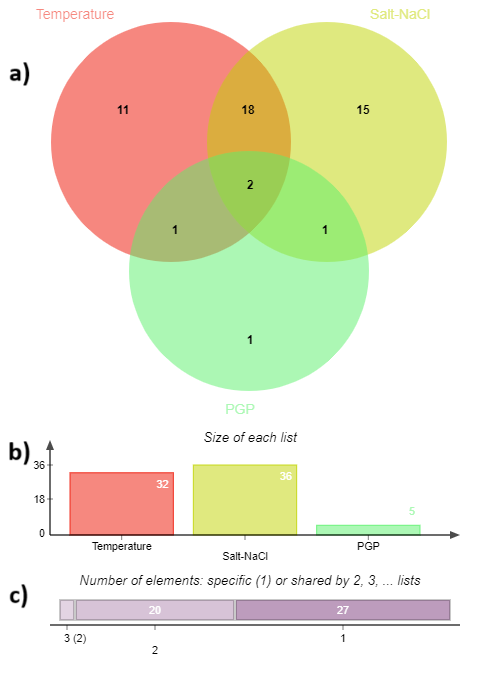


**Figure S3.** Thermotolerant and halotolerant bacterial isolates with multiple plant growth-promoting (PGP) activites a) The isolates that presented each of the possible combinations for the PGP multi-traits, and studied abiotic stresses (i.e. Heat, 50̊ C and Salt, 10% NaCl), b) Size of each studied traits, and c) Number of isolates specific to sharing each of the traits, Values are presented as absolute number of isolates.

**Figure S4.** Effect of bacterial inoculants on shoot length (cm) of germinated seeds of maize measured after 8 days of seed inoculation. Error bars indicate means of shoot length (cm) ± SE. Lettering indicates significance values according to LSD test (*P* ≤ 0.5).

**Figure S5.** Effect of bacterial inoculants on root length (cm) of germinated seeds of maize measured after 8 days of seed inoculation Error bars indicate means of root length (cm) ± SE. Lettering indicates significance values according to LSD test (*P* ≤ 0.5).

**Figure S6.** Effect of bacterial inoculants on the germination (%) of maize seeds after 8 days of seed inoculation Error bars indicate means of germination (%) ± SE. Lettering indicates significance values according to LSD test (*P* ≤ 0.5).
